# A New Optical Remote Sensing Technique for High-Resolution Mapping of Soil Moisture

**DOI:** 10.3389/fdata.2019.00037

**Published:** 2019-11-05

**Authors:** Ebrahim Babaeian, Paheding Sidike, Maria S. Newcomb, Maitiniyazi Maimaitijiang, Scott A. White, Jeffrey Demieville, Richard W. Ward, Morteza Sadeghi, David S. LeBauer, Scott B. Jones, Vasit Sagan, Markus Tuller

**Affiliations:** ^1^Department of Environmental Science, The University of Arizona, Tucson, AZ, United States; ^2^Department of Electrical and Computer Engineering, Purdue University Northwest, Hammond, IN, United States; ^3^School of Plant Sciences, The University of Arizona, Tucson, AZ, United States; ^4^Department of Earth and Atmospheric Science, Saint Louis University, St. Louis, MO, United States; ^5^Department of Civil, Environmental and Geo-Engineering, University of Minnesota, Minneapolis, MN, United States; ^6^Arizona Experiment Station, The University of Arizona, Tucson, AZ, United States; ^7^Department of Plants, Soils and Climate, Utah State University, Logan, UT, United States

**Keywords:** remote sensing, high-resolution, soil moisture, OPTRAM, precision irrigation, TERRA-REF

## Abstract

The recently developed OPtical TRApezoid Model (OPTRAM) has been successfully applied for watershed scale soil moisture (SM) estimation based on remotely sensed shortwave infrared (SWIR) transformed reflectance (TR_SWIR_) and the normalized difference vegetation index (NDVI). This study is aimed at the evaluation of OPTRAM for field scale precision agriculture applications using ultrahigh spatial resolution optical observations obtained with one of the world's largest field robotic phenotyping scanners located in Maricopa, Arizona. We replaced NDVI with the soil adjusted vegetation index (SAVI), which has been shown to be more accurate for cropped agricultural fields that transition from bare soil to dense vegetation cover. The OPTRAM was parameterized based on the trapezoidal geometry of the pixel distribution within the TR_SWIR_-SAVI space, from which wet- and dry-edge parameters were determined. The accuracy of the resultant SM estimates is evaluated based on a comparison with ground reference measurements obtained with Time Domain Reflectometry (TDR) sensors deployed to monitor surface, near-surface and root zone SM. The obtained results indicate an SM estimation error between 0.045 and 0.057 cm^3^ cm^−3^ for the near-surface and root zone, respectively. The high resolution SM maps clearly capture the spatial SM variability at the sensor locations. These findings and the presented framework can be applied in conjunction with Unmanned Aerial System (UAS) observations to assist with farm scale precision irrigation management to improve water use efficiency of cropping systems and conserve water in water-limited regions of the world.

## Introduction and Motivation

Soil moisture is a key hydrologic state variable that links land surface and atmospheric processes (Babaeian et al., 2019). Detailed knowledge about the state of SM and its spatial and temporal dynamics is of crucial importance for crop production to avoid water stress, but also for the mitigation of adverse environmental impacts due to over-irrigation as well as for the conservation of water resources, which is a pressing issue in water-limited regions of the world (Tuller et al., 2019). For example, the water scarcity projections are dire for much of the rapidly growing southwestern United States, for which the Colorado River is the major water source for over 36 million people and for close to 6 million acres of irrigated farmland (Owen, 2017). This escalating water crises demands development and adoption of transformative technologies for precision irrigation management.

Today, airborne remote sensing (RS) techniques with Unmanned Aerial Systems (UAS) provide an exceedingly powerful means for high temporal and spatial resolution SM observations (Stark et al., 2015). However, most of the currently employed RS techniques for SM estimation have been developed and evaluated for coarse-resolution (several tens of kilometers) satellite observations in the optical (Sadeghi et al., 2017), thermal (Shafian and Maas, 2015), and microwave (Kerr et al., 2001; Entekhabi et al., 2010) electromagnetic domains. Despite significant advances in large-scale SM estimation, for example, methods based on microwave observations (e.g., SMAP, SMOS) have limited applicability at smaller scales such as cropped fields, which renders them unsuitable for farm-level precision irrigation management. This advocates the need for the development and implementation of high-resolution RS techniques amenable for field scale SM monitoring and mapping.

Sadeghi et al. (2017) proposed the physically-based OPtical TRApezoid Model (OPTRAM) for estimation of spatiotemporal surface soil moisture dynamics based on the pixel distribution within the Normalized Difference Vegetation Index (NDVI) and shortwave infrared transformed reflectance (TR_SWIR_) space. More specifically, the OPTRAM estimates surface soil moisture based on the physical relationship between TR_SWIR_ and the vegetation cover (Effati et al., 2019). The OPTRAM has been successfully validated with satellite data (i.e., Sentinel-2, Landsat-8, and MODIS) for several watersheds in the U.S. with vastly different climatic conditions, surface topologies, and vegetation covers via comparison of the model's surface soil moisture estimates with ground reference measurements (Sadeghi et al., 2017; Babaeian et al., 2018). Mananze et al. (2019) applied the OPTRAM for agricultural drought monitoring in Mozambique. The advantages of the OPTRAM are twofold—it does not require thermal data such as traditional triangle or trapezoid models, and it can be universally parameterized for a given location because the TR_SWIR_-soil moisture relationship is not affected by ambient environmental factors (e.g., air temperature and wind speed).

Motivated by the successful application of the OPTRAM at the watershed scale, we hypothesize that the model is also applicable to high spatial resolution visible near infrared (Vis-NIR) and shortwave infrared (SWIR) observations as the boundaries of the optical trapezoid are expected to be more distinct for the rather homogeneous surface conditions of agricultural fields when compared to the heterogeneous land surfaces of watersheds. Here, we test this hypothesis with ultrahigh spatial resolution Vis-NIR, and SWIR observations obtained with the TERRA_PHENOTYPING_–REF_ERENCE_ (TERRA-REF) platform, one of the world's largest field phenotyping robots located in Maricopa (AZ), and postulate that if the OPTRAM is applicable for SM mapping with ultrahigh spatial resolution optical reflectance data, it is also amenable for parametrization with high-resolution data captured with UAS's, and thus can be applied for farm scale precision irrigation management.

## Methodology

### Optical Trapezoid Model

**Figure 1:**
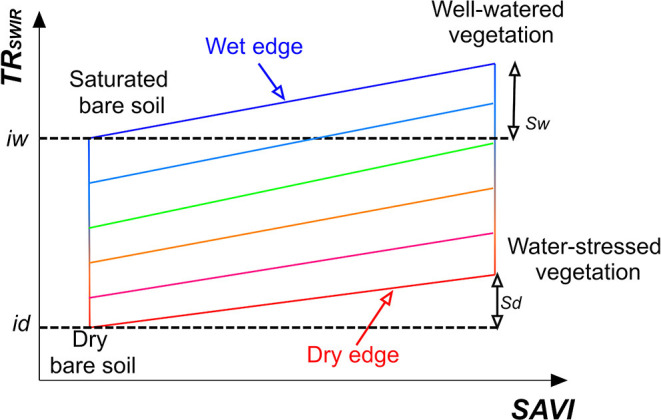
Conceptual sketch of the TR_SWIR_-SAVI feature space.

The volumetric SM content (cm^3^ cm^−3^) is obtained from the trapezoidal geometry of the transformed SWIR reflectance (TR_SWIR_)—vegetation index feature space (Sadeghi et al., 2017) as conceptually shown in Figure 1. For this study, we replaced the previously applied NDVI with the SAVI because it is generally more sensitive to sparse vegetation cover (i.e., the initial and development stages of agricultural crops) as it corrects for the influence of soil brightness when vegetation density is low. This leads to independence of the OPTRAM parameters from soil type. The volumetric SM is calculated as:

(1) $$\begin{array}{l} SM=\frac{i_{dry}+s_{dry}SAVI-TR_{SWIR}}{i_{dry}-i_{wet}+\left(s_{dry}-s_{wet}\right)SAVI}\;\times \;n \end{array}$$

with

(2) SAVI=RNIR - RRedRNIR + RRed + L×(1+L)

and

(3) $$\begin{array}{l} TR_{SWIR}=\frac{{\left(1\;-\;R_{SWIR}\right)}^{2}}{2R_{SWIR}} \end{array}$$

Where *i* and *s* are the intercept and slope of the dry- and wet-edge, respectively (Figure 1), *n* is the soil porosity, L is the soil brightness correction factor, and R_Red_, R_NIR_, and R_SWIR_ denote the reflectances within the Red (636–673 nm), NIR (760–823 nm), and SWIR (2,110–2,290 nm) spectral bands captured with the TERRA-REF Vis-NIR and SWIR imaging systems. The average spectral frequencies of the applicable bands were used to derive R_Red_, R_NIR_, and R_SWIR_. The value of L varies with the extent of the green vegetation cover. For dense vegetation L = 0, and for bare soil L = 1. Generally, L = 0.5 is applied as the default value. When L = 0, then the SAVI equals the NDVI.

### Ultrahigh Resolution Optical Observations and Reference Moisture Measurements

**Figure 2:**
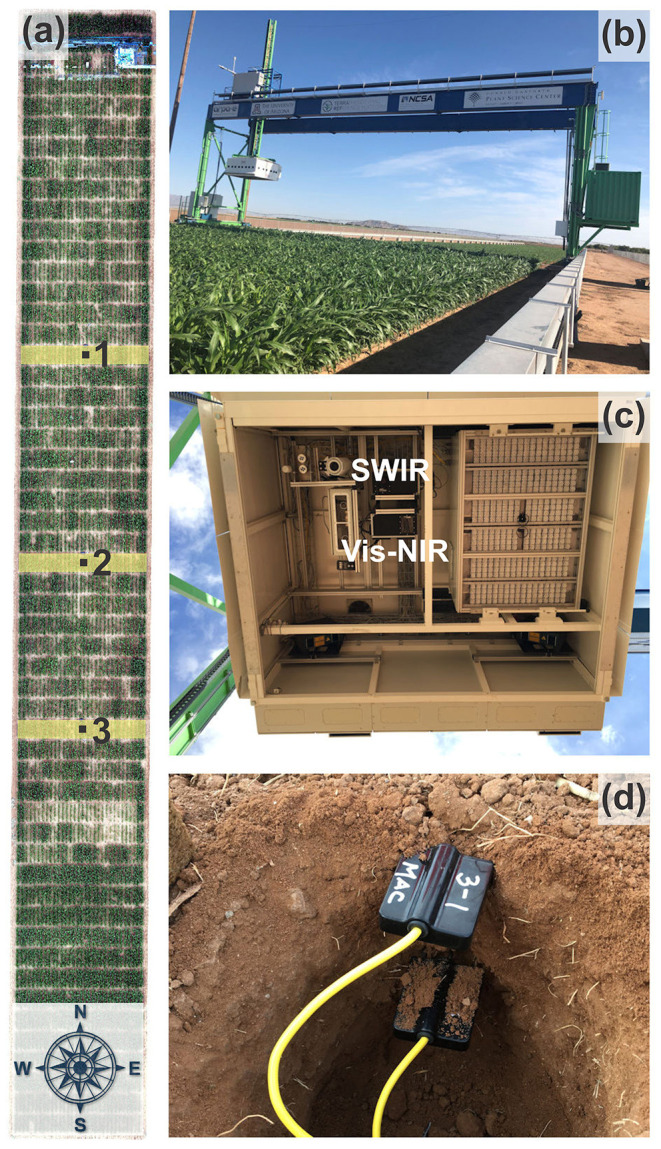
Aerial view of the TERRA-REF field with marked locations (black squares) of the TDR sensor nests and the East-West swats (yellow rectangles) captured by the Vis-NIR and SWIR cameras **(a)**, the TERRA-REF steel gantry with suspended instrument box **(b)**, close up of the instrument box with various imaging systems and sensors **(c)**, and the True TDR-315 sensors installed in duplicate in 2, 10, and 50 cm depths at the three sensor nest locations **(d)**.

The optical observations (i.e., Vis-NIR, and SWIR) for calculation of the SAVI, TR_SWIR_, and SM (Equations 1–3) were acquired at ultrahigh spatial resolution with the TERRA-REF platform (Figure 2a). This state-of-the-art instrument located in Maricopa, Arizona consists of a 30-ton steel gantry with suspended instrument box that contains various imaging systems and sensors, including hyperspectral Vis-NIR and SWIR cameras (Figure 2b) with their specifications listed in Table 1. The robot autonomously moves along two 200-meter steel rails and continuously images the soil and crop below, providing reflectance measurements while the land surface transitions from bare soil to full plant canopy (Burnette et al., 2018).

**Table 1:** Specifications of the TERRA-REF optical imaging systems.

**Imaging System**	**Vis-NIR**	**SWIR**
Imager	Headwall inspector	Headwall inspector
Linear Field of View (FOV) (cm)	100	76
Wavelength (nm)	380–1,000	900–2,500
Pixel Size (mm)	0.6 × 0.6	1.4 × 1.0
Acquisition dates	Sept. 15, Sept. 28, Oct. 9, Oct. 18, Oct. 28, 2018	Sept. 15, Sept. 28, Oct. 9, Oct. 18, Oct. 28, 2018

**Figure 3:**
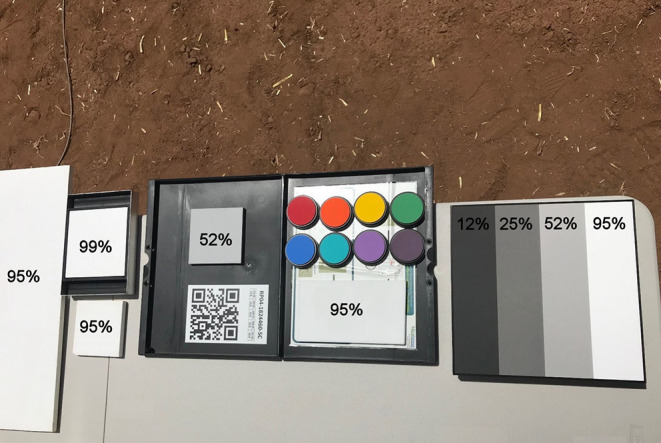
Reference panels exhibiting various spectral reflectances used for radiometric calibration of Vis-NIR and SWIR observations.

The hyperspectral imagers (Headwall Photonics, Bolton, MA, USA) are push-broom sensors that acquire 939 (Vis-NIR) and 275 (SWIR) spectral bands within the 380–1,000 nm and 900–2,500 nm electromagnetic frequency ranges, respectively (Figure 2c). The distance between the soil surface and the hyperspectral cameras was 2-m. The Vis-NIR hyperspectral images for calculation of the SAVI were radiometrically calibrated using real-time down-welling irradiance captured with a spectral irradiance meter (Ocean Optics, Largo, FL, USA) mounted on top of the gantry, which covers the range from 337 to 823 nm. The SWIR images were calibrated with white and gray reference panels with known spectral reflectance values placed at the 2-m distance below the imaging system (Figure 3). Prior to irradiance-based calibration, the dark current was subtracted from the raw image to correct for detector artifacts. Hyperspectral bands over each domain were averaged and used for analysis.

The OPTRAM-estimated SM values were then evaluated based on soil moisture reference measurements with state-of-the-art time domain reflectometry (TDR) sensors (True TDR-315, Acclima, Inc., Meridian, ID, USA) installed at three locations within the scanner field at 2, 10, and 50 cm depths (Figure 2d). The TDR-315 sensor houses the entire measurement circuitry, including a microprocessor, within the sensor head and communicates with a datalogger for transfer of processed data via the SDI-12 protocol. The TDR-measured soil moisture data were recorded at 15-min intervals with CR1000 dataloggers (Campbell Scientific, Inc., Logan, UT, USA). The data used for this study are from an energy sorghum phenotyping experiment conducted from September 1 to October 28, 2018.

## Results and Discussion

### TR_*SWIR*_-SAVI Feature Space for Soil Moisture Estimation With OPTRAM

**Figure 4:**
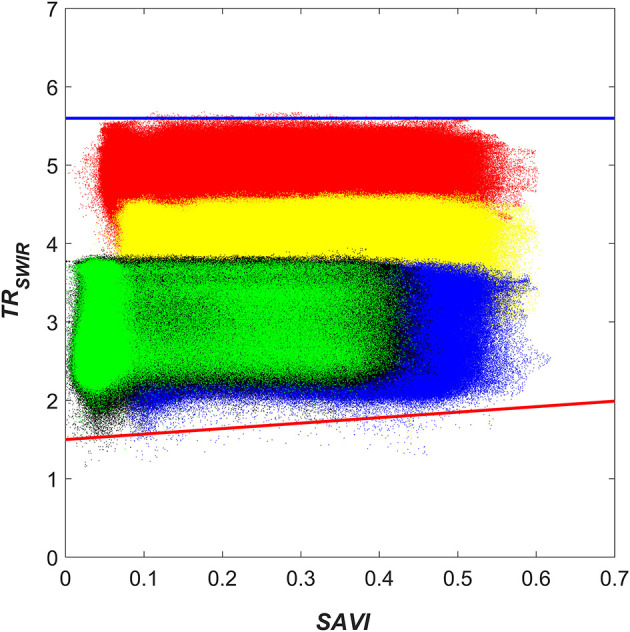
Pixel distribution within the integrated TR_SWIR_-SAVI trapezoidal space for the 2018 sorghum experiment. The blue and red solid lines represent the manually fitted wet- and dry-edges, respectively. The black, green, red, yellow, and blue point clouds correspond to the observations from Sept. 15, Sept. 28, Oct. 9, Oct. 18, and Oct. 28, respectively.

The TR_SWIR_-SAVI trapezoidal space was generated via integration of five scenes collected at the initial stage (i.e., bare soil), during sorghum development, and at the final growth stage (i.e., before harvest) from the beginning of September to the end of October, 2018. The advantages of the integrated trapezoid are increased computational efficiency and independence of dry- and wet-edge parameters from the growth stage. The dry- and wet-edges and their associated parameters (Figures 1 and 4) were determined via manual fitting. This is justifiable because of the very distinct geometry exhibited by the measured TR_SWIR_-SAVI relationship during the sorghum experiment (Figure 4). Furthermore, Babaeian et al. (2018) demonstrated with a thorough sensitivity analysis that the selection of the dry- and wet-edge locations does not need to be overly precise for the OPTRAM to accurately estimate surface soil moisture. It should be noted that the more homogeneous soil and vegetation cover conditions in agricultural fields in conjunction with ultrahigh spatial resolution observations alleviate problems with OPTRAM parameterization occasionally experienced for large scale, coarse-resolution satellite observations, such as oversaturation of the wet-edge or uncertainty associated with image pixels that neither belong to the soil nor to vegetation (e.g., surface water bodies, roads, or buildings) (Sadeghi et al., 2017).

### Soil Moisture Estimation With OPTRAM

**Figure 5:**
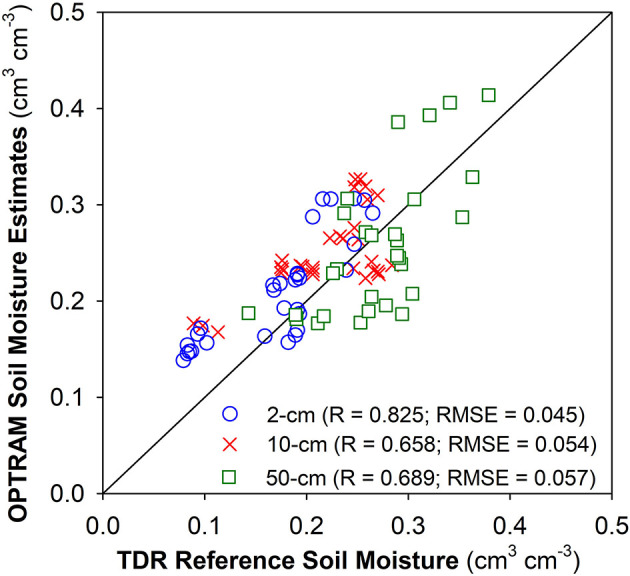
OPTRAM SM estimates compared with the reference TDR measurements at the three sensor nest locations.

To evaluate the accuracy of the OPTRAM SM estimates, they were compared with the TDR reference measurements at the three sensor nest locations. The SM contents measured with the TDR sensors throughout the growth period ranged from 0.029 to 0.465 cm^3^ cm^−3^. The OPTRAM SM was obtained by averaging the pixels directly covering the TDR sensor locations (i.e., an area of 30 times 50 cm). It is interesting to note that because of the ultrahigh resolution, the data volume generated for such a small area with the TERRA-REF Vis-NIR and SWIR imaging systems exceeds several gigabytes for a single scene. Figure 5 depicts the correlation between OPTRAM-determined SM and the TDR reference measurements at 2, 10, and 50 cm depths, with correlation coefficients (R) ranging from 0.66 to 0.83 and root mean square errors (RMSE) from 0.045 to 0.057 cm^3^ cm^−3^, which is considered reasonably accurate for remote sensing of SM (Entekhabi et al., 2014). Because of the limited penetration depth of Vis-NIR and SWIR electromagnetic radiation it was expected that the OPTRAM SM estimates better match the near-surface (2-cm) TDR reference measurements. However, there is also an obvious correlation between the OPTRAM estimates and root-zone SM (Figure 5), which contradicts the common presumption that surface soil moisture is decoupled from the root-zone (Qiu et al., 2014, 2016; Tayfur et al., 2014). Our results support findings in Sadeghi et al. (2019) and Koster et al. (2018), who established links between near-surface SM and the SM in deeper depths, and are in line with results shown in Wu and Dickinson (2004) and Ford et al. (2014), who demonstrate that root-zone SM can be accurately inferred from SWIR remote sensing observations. It is interesting to note that the OPTRAM SM estimates can be applied to directly calculate crop water consumption with a new analytical approach based on inversion of the linearized Richard's equation (Sadeghi et al., 2019). Furthermore, OPTRAM SM time series could be coupled with numerical water flow models to simulate root-zone moisture dynamics considering plant water uptake.

**Figure 6:**
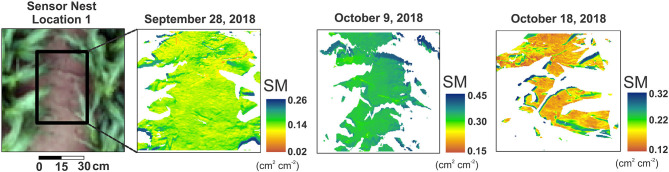
An example for the applicability of the OPTRAM to estimate moisture variations when parameterized with ultrahigh resolution Vis-NIR and SWIR observations. Scenarios for wet and dry conditions are shown.

Figure 6 depicts the OPTRAM soil moisture distributions across sensor nest location 1 at ultrahigh spatial resolution (i.e., 2-mm pixel size) for the early stage of sorghum development (September 28), close to maturity (October 9), and short before harvest (October 18). The maps clearly indicate the OPTRAM's capability to capture soil moisture variations when parameterized with ultrahigh resolution Vis-NIR and SWIR observations, not only for single scenes, but also for various soil moisture states related to irrigation and plant development stage. This instills confidence that the proposed method is amenable for parameterization with high-resolution UAS observations, which opens new avenues for farm scale precision irrigation management to increase crop water use efficiency while alleviating the risk of environmental contamination and contributing to conservation of strained water resources in water-limited regions of the world.

## Conclusions

This contribution should be viewed as a proof of concept for the applicability of the OPtical TRApezoid Model (OPTRAM) for estimation of SM when parameterized with remotely-sensed ultrahigh resolution Vis-NIR and SWIR reflectance data. A comparison of the OPTRAM SM estimates with reference moisture data obtained with state-of-the-art TDR sensors reveals the promising potential of the model to be applied in conjunction with rapidly evolving UAS imaging capabilities. This opens new avenues for UAS-based farm-level precision irrigation management. Such technological advances are much- needed to combat the water crisis in the arid and semiarid regions of the world that is expected to further escalate in view of the rapidly growing human population and a changing global climate. The next step is to test OPTRAM with UAS observations and apply the obtained SM information for farm-level irrigation management.

## Data Availability Statement

The datasets generated for this study are available from the corresponding author on request.

## Author Contributions

EB, MT, MS, and SJ conceptualized the study and contributed to data analyses and writing of the manuscript. SW, MN, JD, RW, and DL contributed to sensor installation, field data collection, and analyses. PS, MM, and VS contributed to the calibration and analyses of the TERRA-REF Vis-NIR and SWIR data.

### Conflict of Interest

The authors declare that the research was conducted in the absence of any commercial or financial relationships that could be construed as a potential conflict of interest.
